# Microbiological Quality of Polish Artisanal Varietal Honeys

**DOI:** 10.3390/foods12183349

**Published:** 2023-09-07

**Authors:** Monika Kędzierska-Matysek, Anna Teter, Tomasz Daszkiewicz, Mariusz Florek

**Affiliations:** 1 Department of Quality Assessment and Processing of Animal Products, University of Life Sciences in Lublin, Akademicka 13, 20-950 Lublin, Poland; monika.matysek@up.lublin.pl (M.K.-M.); anna.teter@up.lublin.pl (A.T.); 2 Department of Commodity Science and Processing of Animal Raw Materials, Faculty of Animal Bioengineering, University of Warmia and Mazury in Olsztyn, Oczapowskiego 5, 10-719 Olsztyn, Poland; tomasz.daszkiewicz@uwm.edu.pl

**Keywords:** honey, origin, physicochemical properties, microbiology, health safety

## Abstract

On the basis of routine microbiological tests and selected physicochemical parameters, the quality and food safety of Polish varietal honeys were evaluated. The study included 21 honey samples from 5 varieties (multifloral, honeydew, rapeseed, buckwheat and linden), in which the moisture and extract content, water activity, pH and free acids were determined, and the colony count, the presumptive *Bacillus* spp., the total fungal count and the presence of anaerobic spore-forming bacilli were examined. More than half (52%, 11/21) of the analyzed honeys contained fewer microorganisms than 10 cfu/g, and in the remaining samples, their numbers ranged from 5 × 10^1^ cfu/g to 4.5 × 10^2^ cfu/g. In all the honeys, the number of presumptive *Bacillus* spp. in 1 g was less than 10 cfu. In 81% (17/21) of the samples, the total count of fungi in 1 g of honey was less than 10 cfu, and the most contaminated was buckwheat honey (3 samples). The anaerobic spore-forming bacteria was detected in 0.1 g only in one sample of buckwheat honey. The values of the physicochemical parameters did not exceed the accepted limits, which indicated that the honey environment was unfavourable for the development of the tested microbial profile.

## 1. Introduction

Consumers perceive honeys as products with special nutritional and health-promoting properties. To meet these expectations, beekeepers should produce high-quality honeys. In addition to physicochemical properties, the key determinant of honey quality is its microbiological state, since these parameters are crucial to the shelf life of the product and food safety for humans.

The antimicrobial properties of honey have been confirmed by many studies. Honey itself has natural mechanisms to inhibit the growth of microorganisms such as bacteria, fungi, viruses and protozoa. The physical barrier is the high osmotic pressure of the honeys, associated with their high sugar content (95–99% of honey’s dry weight), as well as their low pH (3.2–4.5). Chemical compounds, i.e., hydrogen peroxide, high content of thermostable polyphenols (phenolic acids and flavonoids), methylglyoxal MGO (in manuka honey), amino acids, ascorbic acid, terpenes, benzyl alcohols and benzoic acid make honeys active against bacteria. In contrast, antimicrobial biological agents including peptides, i.e., lysozyme and defensin-1 are present in products containing pure medical honey, such as Revamil balm [1,2,3,4,5]. In this regard, the results reported by Hossain et al. [6] are optimistic, indicating that such preparations maintain the antimicrobial activity of the honeys during the entire process of formulation for therapeutic use.

The microbiological profile of honey is influenced by its physicochemical properties, in particular its water content, acidity, the presence of sugars and natural antimicrobial substances [3]. Among the microbiota present in honey, two orders of bacteria predominate, i.e., *Lactobacillales* and *Bacillales*, with a significant proportion (60–90%) of all microbes in honey within *Bacillales* being the genera *Bacillus* and *Paenibacillus* [7]. Honey is also colonized by mould fungi, which predominate in honeydew honeys, and yeast fungi (genus *Saccharomyces*, *Torulopsis*), which predominate in nectar honeys [8]. A characteristic feature of fungi is their production of antimicrobial compounds, i.e., antibiotics and toxins (mycotoxins). Mycotoxins have an inhibitory and even bactericidal effect on competing microorganisms found in honey. Lactic acid bacteria and *Bacillus* spp. present in honey have been shown to have a strong enzyme system that converts mycotoxins to non-toxic derivatives. Although mycotoxins (e.g., aflatoxins) are not present in unprocessed honey [9,10]. Another biological threat found in honey is microorganisms that produce spores resistant to adverse environmental conditions. Among them, Gram-positive anaerobic spore-producing *Clostridium*, aerobic spore-producing bacilli and moulds are detected [11,12]. Spores can survive in honey for a long time, withstanding ambient (environmental) temperatures and thermal processes. The antimicrobial effect of honey plays a role in reducing the population of microorganisms and, consequently, spores to a minimal number in honey [13]. It is generally accepted that a small number of low-diversity microbiota are present in honey [14]. The phenomenon of spores not converting to vegetative forms and remaining alive in honey indicates its inhibitory role against microorganisms [4,12].

Microbial contaminants can enter honey through various routes, such as the raw materials used in its production (plant nectar, secretions of living plant parts, insect excretions and pollen). The bees themselves can also contaminate the product, especially if they are sick. Environmental components such as soil, air and water are other sources of honey contamination. The level of microbial contamination can also be influenced by humans and their actions, including the environment and equipment used during product manufacturing. Secondary sources of contamination, to a large extent, can be controlled by following good production practices in beekeeping [9,15].

In the European Union, there is a lack of official permissible levels of microbial contamination for honey and the reference microbiological tests have not been established. Therefore, the aim of the study was to determine the food safety of Polish artisanal nectar and honeydew honeys on the basis of routine microbiological tests and selected physicochemical parameters.

## 2. Materials and Methods

### 2.1. Honey

The study included 21 honeys obtained directly from artisanal apiaries located in the Lublin region (an area of south-eastern Poland). Lubelskie Voivodeship is the third largest province in Poland (accounting for 8% of the country’s area). Favorable soil and climate conditions (soil quality, water conditions, agroclimate and relief) make Lubelskie one of the three most important regions in the country characterized by the leading role of the agricultural sector, with Lubelskie having the highest share of agricultural land in the total area (70%) compared to other regions. The average forest cover of the region is about 25% (slightly lower than the national average), which is related to the high fertility of soils, with a preference for their agricultural use. The geographical origin of honeys was described in detail by Kędzierska-Matysek et al. [16]. The honeys were stored in airtight glass jars, without exposure to sunlight at a room temperature of 20 °C (±2 °C). The percentage of dominant pollen grains in honey samples was determined according to the Regulation of the Ministry of Agriculture and Rural Development [17]. Based on the proportion of dominant pollen, 5 varieties were distinguished, i.e., multifloral (without guiding pollen, MF—8 samples), rapeseed (*Brassica napus* L., RS—3 samples), buckwheat (*Fagopyrum esculentum* Moench, BW—4 samples), linden/lime (*Tilia* spp. L., LI—3 samples) and honeydew (honeydew, HD—3 samples) honeys.

### 2.2. Analyses

#### 2.2.1. Physicochemical Measurements

The percentage of water and extract content (% m/m) in the decrystallized honeys was determined from the refractive index read with an Abbe refractometer (Carl Zeiss, Jena, Germany), taking into account a correction for the refractive index of 0.00023 for each degree of temperature above 20 °C [17]. Water activity (a_W_) in undiluted honeys was determined using a HygroLab C1 Rotronic instrument with an HC2-AW probe (Bassersdorf, Switzerland). Measurements were performed in AWQ mode with 15-min stabilization. Before testing, honey samples were conditioned at room temperature (20 ± 1 °C).

The specific conductance was determined by measuring the electrical resistance of the honey solution (20 g in 100 mL of distilled water on a dry weight basis). The measurement was made using a Pioneer 65 Meter (Radiometer Analytical, Villeurbanne, CEDEX-France) with a conductivity chamber (CDC 30T). The result was expressed in millisimens per centimetre (mS/cm) [17].

The pH was determined in an aqueous solution of the honey sample, prepared by dissolving 10 g of honey weighed to the nearest 0.001 g in 75 mL of carbon dioxide-free distilled water. In the next step, the solution thus prepared was titrated with a 0.1 M NaOH solution to pH 8.30 to determine free acidity (N = mL 0.1 M NaOH × 10) expressed in mval/kg. Both parameters were measured using a Pioneer 65 Meter (Radiometer Analytical, Villeurbanne, CEDEX-France) with a conductivity chamber (CDC 30T).

#### 2.2.2. Microbiological Tests

The honeys were subjected to commercial microbiological testing at one of the national reference laboratories (NVRI, Report No: P/19/53936/ZHS/2426M-2446M), including colony count at 30 °C by the surface plating technique according to PN-EN ISO 4833-2:2013-12+AC:2014 Ch.2. [18], isolation and enumeration of presumptive *Bacillus* spp. according to PN-EN 15784:2009 [19], enumeration of total fungal count and detection of the presence of anaerobic spore-forming bacilli in 0.1 g, both in accordance with PN-R-64791:1994 [20]. For the quantitative methods used, the uncertainty of the result was estimated, which did not include values below the limit of quantification. For the qualitative method, an uncertainty budget of measurement was determined. Microbial counts were expressed as colony-forming units per gram of honey (cfu/g).

### 2.3. Statistical Analysis

The obtained data were analyzed statistically using Statistica ver. 13 software (TIBCO Software Inc., Palo Alto, CA, USA). The effect of honey variety (multiflower, rapeseed, buckwheat, linden or honeydew) on physicochemical properties was verified using one-way ANOVA, and Duncan’s test was used to compare mean characteristics.

## 3. Results

### 3.1. Physicochemical Properties

There was a significant effect of honey variety on all the evaluated physicochemical properties (Table 1).

**Figure 1:**
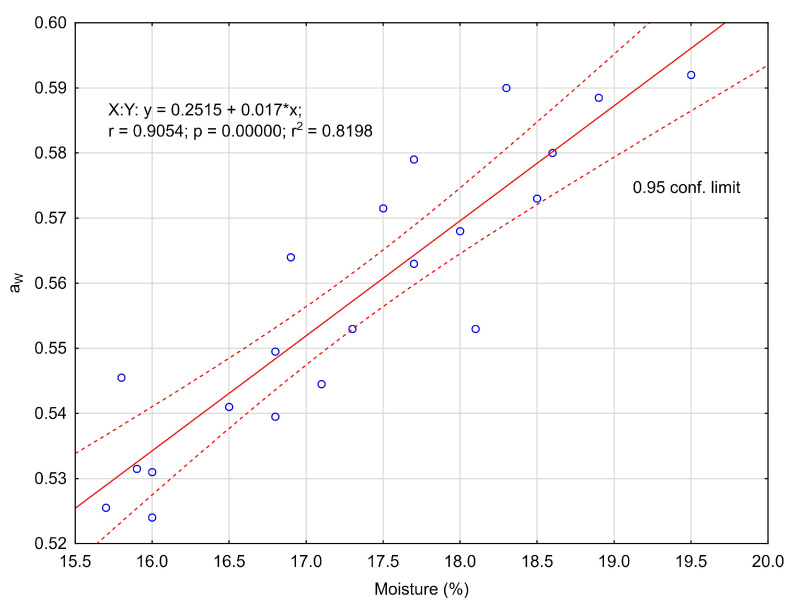
Moisture content (%) and water activity relationship in honey.

Significantly (*p* < 0.05), honeydew honey contained the least water (average 16.33%), while buckwheat and rapeseed honey contained the most (18.23% and 18.10%, respectively). In food analytics, the extract is assumed to be the sum of water-soluble substances and non-volatile compounds with water vapor. Since extract content is equivalent to water content, honeydew honey contained significantly (*p* < 0.05) the most extract (average 81.93%), and buckwheat and rapeseed honeys contained the least (80.08% and 80.20%, respectively). Buckwheat and rapeseed honeys showed significantly the lowest pH (3.66 and 3.80, respectively), while the highest pH was found in honeydew honey (4.16). Variety significantly (*p* < 0.05) differentiated the degree of acidity of the honeys, which was between 21.5 mval kg^−1^ (RS) and 47.3 mval kg^−1^ (BW). The other varieties had similar acidity (32.8–35.7 mval kg^−1^). The significantly (*p* < 0.05) highest average water activity was detected in rapeseed honey (a_W_ = 0.575) and buckwheat honey (a_W_ = 0.574), which also contained the most water, in relation to honeydew honey (a_W_ = 0.575) with the lowest water activity and at the same time the least water content. The results obtained here (Figure 1) confirm a significant linear relationship (r = 0.905) between these two parameters.

### 3.2. Microbiology Criteria

The total colony count in 52% (11/21) of the tested honeys was less than 10 cfu/g (Table 2). In the remaining 48% (10/21) of samples, microbial contamination was higher, ranging from 5.0 × 10^1^ cfu/g to 4.5 × 10^2^ cfu/g. Taking this criterion into account, the honeys can be ranked as follows (from the smallest to the largest number of microorganisms content): linden—multiflower—rapeseed—honeydew—buckwheat. No cells of presumptive *Bacillus* spp. bacteria were found in all honey samples regardless of variety.

Fungal microflora contamination was detected in four samples, including two buckwheat honeys, with 2.0 × 10^1^ cfu/g each, and two samples (buckwheat and honeydew) where there were less than 40 cfu/g. In the remaining 17 samples (81%), the total count of fungi in 1 g of honey was less than 10 cfu. In addition to vegetative forms, the presence of anaerobic spore-forming bacilli was tested in the honey samples evaluated. Spores of these bacteria were detected in only one sample (in 0.1 g of buckwheat honey).

Summarizing the obtained results, it can be claimed that linden honeys turned out to be the cleanest microbiologically, as the total number of microorganisms, fungi and presumed *Bacillus* spp. did not exceed 10 cfu/g, and no anaerobic spore bacilli were detected in 0.1 g. Buckwheat honeys, on the other hand, were the most contaminated. In particular, sample No. 15 contained the highest number of microorganisms (4.5 × 10^2^ cfu/g), fungi (2.0 × 10^1^ cfu/g) and anaerobic spore-forming bacilli per 0.1 g.

## 4. Discussion

Water content and activity are the main indicators of honey, which affect the survival and multiplication of bacteria in it [21]. Too much water content in honey promotes its fermentation (by sugar-tolerant yeast) [22]. In turn, the high concentration of sugar binds water molecules, thus bacteria have limited availability of water necessary for their growth. In addition, sugar exerts osmotic pressure, making bacterial cells dehydrated by osmosis and unable to live in a hypertonic sugar solution [1]. On the basis of the present study, it was confirmed that none of the tested samples of 21 honeys exceeded the water content allowed in EU countries (<20%) [23].

An indicator of the potential use of water by microorganisms is water activity. A linear relationship has been shown between moisture content and water activity, i.e., with an increase in moisture content there is an increase in water activity, and vice versa [24,25]. Bakier [24] revealed that for the same water content, rapeseed honey showed higher water activity compared to buckwheat honey. This phenomenon was related to the stronger binding of water molecules in the glucose solution. In addition, Bakier [24] identified the threshold level of water activity (a_W_ = 0.6) for buckwheat honey at a moisture content of 19.83%. If the moisture content of the honey is increased above this critical level and the temperature is higher than 25 °C, fermentation due to the activity of osmophilic yeast can begin [26]. Therefore, the water activity below 0.6 ensures the stability of honey during customary storage by consumers at ambient temperature. The water activity of all evaluated individual honey samples in the presented studies ranged from 0.524 to 0.592, i.e., below the critical level indicated in the literature. Due to the low water activity of undiluted honeys (a_W_ = 0.6), bacteria do not grow in this product (the limiting a_W_ range for bacterial growth is 0.91 to 0.98, for yeast fungi 0.88 and mould 0.80, and xerophilic mould 0.65) [2]. If the critical value of a_W_ is exceeded in honey, osmophilic yeast may develop and ferment during storage [24]. National research results in this area [27] showed that the pH of linden honey (from Polish apiaries) from the Warsaw market retail chain was lower (3.37–4.09), water activity was similar (0.53–0.57), and water content was more variable (14.9–19.1%) compared to the presented results for the same honey variety. The differences presented between our own and the cited results, especially with regard to the low pH of the honey, may have been due to the longer time between obtaining the honey from the apiary and performing the analyses. It has been shown that during storage (regardless of temperature) the free acidity of honey increases and its pH decreases [28]. In the present study, the honeys came directly from the beekeepers, while in the studies [25], the honeys from the apiaries were purchased from one of the retail networks, so it is difficult to assess exactly how long the distribution took.

Water activity also depends on water-glucose interactions that lead to crystallization [29]. A combination of factors, such as low values of water activity, pH, and protein content at high osmotic pressure, inhibit bacterial growth in honey [30]. Xiong et al. [31] indicate that the fungal community (population) (measured by Jaccard distance) is significantly related to water activity and honey colour. In the presented study, the water activity of all honey samples was below 0.6 (Table 1).

The acidity of honey, expressed in terms of honey reaction (pH) and total acidity (in mval kg^−1^), affects the quantitative and qualitative composition of microorganisms. Due to the high content of sugars in honey, its acidity is masked. The high acidity of honey is related to the occurrence of various organic acids, amino acids and phenolic acids [2], with gluconic acid being of particular importance [32]. Honey has a low pH (3.2 to 4.5). Such a range is sufficient to inhibit the growth of pathogenic microorganisms, such as *Streptococcus* pyogenes (pH 4.5), *Escherichia coli* (pH 4.3), *Pseudomonas aeruginosa* (pH 4.4) and *Salmonella* spp. (pH 4.0) [1,33]. The honeydew honeys evaluated in this study showed a pH from 3.51 to 4.2, and one sample of honeydew honey showed a pH = 4.52. As far as free acidity is concerned, despite its variability in individual honey samples (i.e., from 16 mval kg^−1^ in multifloral honey to 49 mval kg^−1^ in buckwheat honey), was not higher than the limit of 50 mval kg^−1^ adopted in the EU countries [23] (Table 1). Dobrina et al. [34] reported free acidity of monofloral acacia honey from different sources between 18.9 and 40.2 meq kg^−1^.

The microbiological quality of honeys can be determined by conducting routine microbiological tests for groups of microorganisms that act as hygienic indicators, such as total microbial counts, fungal counts, numbers of putative *Bacillus* spp. and anaerobic spore-forming bacilli. *Bacillus* spp. are Gram-positive bacteria that form heat-resistant spores. Since they are stable in acidic environments, they can colonize honey [35]. The obtained results of microbiological tests of honeys in the presented studies cannot be referred to the EU legislation, because the official permissible levels of microbial contamination for this product have not been established [15]. The results obtained from our own research will be discussed with those of national authors. Rosiak et al. [26] reported a wider range of microbial contamination of Polish linden honey available in the retail network of the city of Warsaw by aerobic mesophilic microorganisms (log 0.74–2.07 cfu/g) according to the classical method of depth culture than in the presented study (<10 cfu/g). Recently, Ziuzia et al. [36] isolated from Polish linden honeys 15 wild-type yeast strains, which belonged to such species as *Candida magnoliae*, *Yarrowia lipolytica*, and *Starmerella magnoliae*. According to Snowdon and Cliver [37], despite the initial low yeast content in various honeys (less than 100 cfu/g), unfortunately, the level can increase dramatically later. In the present study, the highest total count of fungi in different artisanal honeys did not exceed 40 cfu/g.

Most bacteria of the genus *Bacillus* are not harmful to mammals, apart from the likes of *B. cereus* and *B. anthracis*. *B. cereus* is classified as a particularly dangerous pathogen, which is an infectious agent that is foodborne [15]. *Bacillus* spp. produce functional antibiotics (bacitracin, bacilysin), siderophores, lipopeptides (iturin, surfactin, phengycin and bacillomycin), bacteriocins (subtilisin, subtilosin, lichenicidin, lichens, thuricins and cereins), which are antimicrobial compounds. Therefore, these biological characteristics make them probiotic bacteria [5,6,7,8,9]. Pomastowski et al. [15] indicate that, irrespective of geographic and botanical origin, the tested honeys were dominated by spore-forming *Bacillus* spp. At the same time, the proportion of *B. cereus* in samples of Polish honeys was over 4 times lower than in samples from abroad. Furthermore, irrespective of the country of origin, most of the identified *B. cereus* were detected in multi-flower honeys (5/9). On the other hand, on the basis of their PCA analysis, they showed that *B. cereus* group bacteria were most prevalent in honeys with higher pH and lower acid content. When examining Polish acacia honey, Rosiak et al. [27] detected in two out of 7 samples *Bacillus* spp. spore-forming bacteria at levels above 5 × 10^2^ cfu/g. In the remaining 5 samples microbial contamination was higher than 100 cfu/g.

Although honey is recognized as one of the safer foods from a microbiological point of view, spores can survive even in such a hostile environment as honey [30]. Good quality honeys show an a_W_ below 0.6, while to sustain *Clostridium botulinum* proliferation and botulinum toxin production the a_W_ value should be higher than 0.9 [38]. Botulinum toxin is the aetiological agent of botulism. The human lethal dose ranges from 0.2 µg/kg to 2 µg/kg. Children between the ages of 2 weeks and 1 year are particularly vulnerable to botulism, whose immature gut microbiome allows ingested spores to germinate, multiply and consequently produce botulinum neurotoxins [39]. The results indicate a low contamination of the tested honeys with anaerobic spore-forming bacilli. Out of 21 honeys, spores were found in only one sample (in 0.1 g of honey). Wojtacka et al. [40] examined a total of 102 honey samples collected from small apiaries (20 hives) in Poland showing that 22 (21.6%) samples were contaminated with *C. botulinum* spores of types A, B and E. Grenda et al. [39], after examining 240 samples of multiflower honey collected from Polish apiaries, found the occurrence of *C. botulinum* type A and B strains in 5 (2.1%) honey samples.

## 5. Conclusions

The values of the physicochemical parameters tested did not exceed the required ranges for moisture content (<20%), water activity (<0.6), free acidity (<50 mval kg^−1^) and low pH, indicating the honey environment was unfavourable for the development of the microbial profile tested and the stability of the product. In all honey samples, irrespective of the variety, no cells of the putative *Bacillus* spp. bacteria were found. Most microbiological contaminants were found in buckwheat honey among the 5 varieties evaluated. The presented results testify to an acceptable bacteriological quality of Polish honeys, with the lowest level of microbiological contamination in linden honeys. Nevertheless, our results are only a contribution to further extended research especially in the field of microbiology, as a result of which it will be possible to establish certain microbiological criteria and limits to ensure the food safety of honeys.

## Figures and Tables

**Table 1:** Physicochemical properties of the honey types.

No	Origin Type of Honey	Moisture (%)	Extract (%)	pH	Free Acidity (mval kg^−1^)	a_W_
1	MF	16.8	81.5	3.72	48.0	0.539
2	MF	15.8	82.4	3.69	42.0	0.545
3	MF	17.1	81.2	4.16	38.0	0.544
4	MF	17.7	80.6	3.93	34.0	0.563
5	MF	16.0	82.2	3.73	36.5	0.524
6	MF	17.7	80.6	3.79	25.0	0.579
7	MF	18.1	80.2	3.99	27.0	0.553
8	MF	18.3	80.0	4.01	16.0	0.590
	mean ± s.d.	17.19 ±0.93 ^ab^	81.09 ± 0.89 ^ab^	3.88 ± 0.17 ^ab^	33.31 ± 10.23 ^ab^	0.555 ± 0.022 ^ab^
9	HD	15.7	82.5	4.52	32.0	0.525
10	HD	16.5	81.8	3.93	41.0	0.541
11	HD	16.8	81.5	4.04	34.0	0.549
	mean ± s.d.	16.33 ± 0.57 ^a^	81.93 ± 0.51 ^b^	4.16 ± 0.31 ^b^	35.67 ± 4.73 ^bc^	0.539 ± 0.012 ^a^
12	RS	18.5	79.8	3.80	27.0	0.573
13	RS	16.9	81.4	3.68	19.5	0.564
14	RS	18.9	79.4	3.92	18.0	0.588
	mean ± s.d.	18.10 ± 1.06 ^b^	80.20 ± 1.06 ^a^	3.80 ± 0.12 ^a^	21.50 ± 4.82 ^a^	0.575 ± 0.012 ^b^
15	BW	17.5	80.8	3.87	48.7	0.571
16	BW	18.6	79.7	3.51	46.5	0.580
17	BW	17.3	81.0	3.68	45.0	0.553
18	BW	19.5	78.8	3.58	49.0	0.592
	mean ± s.d.	18.23 ± 1.02 ^b^	80.08 ± 1.02 ^a^	3.66 ± 0.16 ^a^	47.30 ± 1.90 ^c^	0.574 ± 0.016 ^b^
19	LI	16.0	82.2	4.20	21.5	0.531
20	LI	15.9	82.3	3.82	42.0	0.531
21	LI	18.0	80.3	3.91	35.0	0.568
	mean ± s.d.	16.63 ± 1.18 ^ab^	81.60 ± 1.13 ^ab^	3.98 ± 0.20 ^ab^	32.83 ± 10.42 ^ab^	0.544 ± 0.021 ^ab^
	Total	17.31 ± 1.11	80.96 ± 1.07	3.88 ± 0.23	34.56 ± 10.58	0.557 ± 0.022

a_W_, water activity; MF, multifloral; HD, honeydew; RS, rapeseed; BW, buckwheat; LI, linden; s.d., standard deviation. Means with different letters ^a,b,c^ in rows differ significantly according to the Duncan’s test at *p* < 0.05.

**Table 2:** Microbiological criteria of honey types.

No	Origin Type of Honey	Total Count of Fungi (cfu/g)	Presumptive *Bacillus* spp. (cfu/g)	Anaerobic Spore-Forming Bacteria (in 0.1 g)	Total Bacteria Count (cfu/g)
1	MF	<10	<10	n.d.	<10
2	MF	<10	<10	n.d.	3.0 × 10^2^
3	MF	<10	<10	n.d.	<10
4	MF	<10	<10	n.d.	<10
5	MF	<10	<10	n.d.	5.0 × 10^1^
6	MF	<10	<10	n.d.	<10
7	MF	<10	<10	n.d.	<10
8	MF	<10	<10	n.d.	5.0 × 10^1^
9	HD	<40	<10	n.d.	1.5 × 10^2^
10	HD	<10	<10	n.d.	2.5 × 10^2^
11	HD	<10	<10	n.d.	5.0 × 10^1^
12	RS	<10	<10	n.d.	<10
13	RS	<10	<10	n.d.	5.0 × 10^1^
14	RS	<10	<10	n.d.	3.5 × 10^2^
15	BW	2.0 × 10^1^	<10	detected	4.5 × 10^2^
16	BW	<40	<10	n.d.	<10
17	BW	2.0 × 10^1^	<10	n.d.	2.5 × 10^2^
18	BW	<10	<10	n.d.	<10
19	LI	<10	<10	n.d.	<10
20	LI	<10	<10	n.d.	<10
21	LI	<10	<10	n.d.	<10

cfu, colony forming unit; MF, multifloral; HD, honeydew; RS, rapeseed; BW, buckwheat; LI, linden; n.d., not detected.

## Data Availability

The microbiological data presented in this study (NVRI, Report No: P/19/53936/ZHS/2426M-2446M) are available on request from the corresponding author.
